# Occurrence of Pathogenic and Allergenic Molds in the Outdoor and Indoor Environment of a Major Hospital and Molecular Epidemiology of *Aspergillus fumigatus* in Kuwait

**DOI:** 10.3390/jof11020083

**Published:** 2025-01-21

**Authors:** Mohammad Asadzadeh, Suhail Ahmad, Ferry Hagen, Jacques F. Meis, Ziauddin Khan

**Affiliations:** 1 Department of Microbiology, Faculty of Medicine, Kuwait University, Safat 13110, Kuwait; mohammad.assadzadeh@ku.edu.kw (M.A.); ziauddin381044@gmail.com (Z.K.); 2 Department of Medical Mycology, Westerdijk Fungal Biodiversity Institute, 3584 CT Utrecht, The Netherlands; 3 Institute for Biodiversity and Ecosystem Dynamics (IBED), University of Amsterdam, 1098 XH Amsterdam, The Netherlands; 4 Department of Medical Microbiology, University Medical Center Utrecht, 3584 CS Utrecht, The Netherlands; 5 Radboudumc—CWZ Center of Expertise for Mycology, 6525 GA Nijmegen, The Netherlands; j.meis@radboudumc.nl; 6 Institute of Translational Research, Cologne Excellence Cluster on Cellular Stress Responses in Aging-Associated Diseases (CECAD) and Excellence Center for Medical Mycology, University of Cologne, 50923 Cologne, Germany

**Keywords:** *Aspergillus fumigatus*, environmental molds, isolation, triazole resistance detection, microsatellite typing

## Abstract

Aspergilli and other molds are prevalent in the environment and are an important cause of opportunistic infections and seasonal allergies in susceptible patients. This study determined species distribution of various molds in outdoor/indoor air in and around a major hospital and performed antifungal susceptibility testing and molecular fingerprinting of environmental and clinical *Aspergillus fumigatus* isolates in Kuwait. Sampling for the isolation of molds was performed for a 17-month-period from the water/indoor air of medical/surgical wards/ICUs and outdoor air. Molds were identified by phenotypic characteristics and/or by the PCR-sequencing of rDNA/β-tubulin/calmodulin genes. Antifungal susceptibility testing was done by Etest. Fingerprinting was performed by nine-loci-based microsatellite analysis. A total of 6179 isolates were obtained from outdoor (*n* = 4406) and indoor (*n* = 1773) environments. These included *Cladosporium* spp. (*n* = 2311), *Aspergillus* spp. (*n* = 1327), *Penicillium* spp. (*n* = 1325), *Paecilomyces* spp. (*n* = 473), *Alternaria* spp. (*n* = 218), *Bipolaris* spp. (*n* = 133), and other molds (*n* = 392). Fingerprinting data revealed heterogeneity among clinical and environmental *A. fumigatus* and shared genotypes among outdoor air and hospital environmental isolates. Itraconazole-resistant *A. fumigatus* isolates with TR_34_/L98H mutations in *Cyp51A* were also recovered from outdoor air (*n* = 1), a hospital environment (*n* = 3), and clinical samples (*n* = 2). More than 15 fungal genera and all four *Aspergillus* (*Nigri*, *Flavi*, *Fumigati*, and *Terrei*) sections and nine rare aspergilli were detected. The isolation frequency was higher during the peak allergy season of October/November. The presence of shared genotypes among outdoor air and the hospital environment including triazole-resistant *A. fumigatus* suggests a reservoir for invasive infections among susceptible hospitalized patients.

## 1. Introduction

Invasive fungal infections (IFIs) are increasing worldwide due to an expanding population of hospitalized patients with compromised or suppressed immunity and multiple other comorbidities as well as due to the emergence of drug-resistant/novel fungal pathogens and are usually associated with high mortality rates [1,2,3,4,5,6]. *Aspergillus* spp., *Cladosporium* spp., *Penicillium* spp., *Fusarium* spp., mucorales, and other molds are nearly universally present in soil and ambient air. These molds grow on damp or decaying organic matter and water reservoirs in homes and healthcare facilities, and some of these molds are also important human pathogens, particularly for patients with compromised or suppressed immunity [7,8,9].

*Aspergillus* spp. cause diseases ranging in severity from life-threatening invasive pulmonary aspergillosis (IPA) to less severe chronic pulmonary aspergillosis (CPA) and allergic bronchopulmonary aspergillosis (ABPA) in susceptible individuals [8,10,11,12,13,14]. Studies have shown that previous sensitization with *Aspergillus* antigens varies from 16% to 38% in asthmatic subjects in different geographical regions of the world [15]. *Aspergillus fumigatus* is the most common causative agent; however, other species such as *A. flavus*, *A. terreus*, *A. niger*, and other *Aspergillus* spp. can also cause disease in susceptible patients [13,16,17,18,19]. Although IPA typically involves immunocompromised individuals, particularly those with prolonged neutropenia, recent studies have highlighted the involvement of other at-risk populations such as patients with acute influenza and SARS-CoV-2 infections [20,21,22]. Triazoles like itraconazole, posaconazole, voriconazole, and isavuconazole, are highly active against *A. fumigatus* and other *Aspergillus* spp. and are used as first-line therapy in the management and prophylaxis of IPA [22,23,24]. However, therapy with triazoles for long durations induces the emergence of drug-resistant aspergilli mainly due to single nucleotide changes in the *cyp51A* gene, which encodes 14α-sterol demethylase [25,26,27,28]. Resistance to triazoles also develops in aspergilli in the environment due to their exposure to azole fungicides that are being used extensively for crop plant protection [25,26,27,28]. The most commonly acquired and environmentally derived mechanism of resistance involves the presence of a tandem repeat of 34 nucleotides (TR_34_) in the promoter region together with a nonsynonymous mutation (L98H) in *cyp51A*. Patients can also acquire azole-resistant *A. fumigatus* from the environment carrying TR_34_/L98H mutations [25,26,27,28].

Kuwait, a small Arabian Gulf country in the Middle East, has witnessed unprecedented plantation drives to make this arid/desert country green and massive construction projects in the last three decades, and both these activities are also associated with higher spore counts of common molds, including drug-resistant strains in the outdoor/indoor air [7,8,9,27,29,30,31]. Although healthcare facilities housing immunocompromised patients employ various strategies to minimize the exposure of susceptible patients to fungal spores such as high-efficiency particulate air (HEPA) filters, studies have shown that it is nearly impossible to completely eliminate pathogenic molds from indoor hospital environments [7,8,9,13,14,17,20,21,22]. Only scant information is available on the occurrence of pathogenic molds in outdoor and indoor hospital environments in Kuwait. In two preliminary studies, the presence of *Aspergillus calidoustus* and triazole-resistant *A. fumigatus* has been described both outdoors and in hospital environments [32,33]. *A. fumigatus* with environmentally derived triazole-resistance due to TR_34_/L98H or TR_46_/Y121F/T289A mutations have been isolated from clinical samples [34,35]. This study was carried out to determine the prevalence of various molds in outdoor/indoor air in and around a major hospital. Phenotypic characteristics and multigene sequencing were used for the identification of rare molds, and antifungal susceptibility testing and molecular fingerprinting of environmental and clinical *A. fumigatus* isolates were also carried out.

## 2. Materials and Methods

### 2.1. Environmental Sampling, Clinical Samples and Culture

Outdoor and indoor air sampling was performed at ground level and at 15 m above the ground level. Additionally, water samples were collected from indoor water taps, while cotton swabs were used to obtain samples from wash basins, floors, and air-conditioning ducts of different wards and intensive care units (ICUs) of a major hospital (Hospital A) over a 17-month (October 2011 to February 2013) period. All samples were processed for the growth of fungi by evenly spreading on the surface of malt extract agar (MEA) plates, as described previously [33]. The plates were incubated at 30 °C for up to 7 days, and the number of fungal colonies (colony forming units, CFUs) was counted. Cultures were also grown at 37 °C and 42 °C to determine thermotolerance of rare aspergilli detected in this study. Clinical *A. fumigatus* isolates were recovered from various specimens of hospitalized patients and were available from the culture collection of Mycology Reference Laboratory within the Department of Microbiology, Faculty of Medicine, Kuwait University. A total of 117 *A. fumigatus* isolates collected from 1991 to 2018 were used for in vitro antifungal susceptibility testing. The clinical specimens yielding these isolates are listed in Supplementary Table S1. This study was approved by the Ethical Committee of Health Sciences Center, Kuwait University (Approval no. VDR/EC-386 dated 31 May 2023). Fungal isolates were identified by phenotypic characteristics and/or by molecular methods [33,36].

### 2.2. DNA Isolation and Molecular Identification

The DNA from the isolates was prepared as described in detail previously [37]. Briefly, each isolate was freshly grown in liquid culture or on the surface of a Petri plate, and the pelleted cells or mycelial mat were transferred into 50 mL polypropylene screw cap tubes containing six glass beads (4 mm diameter). The tubes were immersed in liquid nitrogen for 10 s and vortexed vigorously for 30 s. The DNA was then extracted by using phenol-chloroform-isoamylalcohol, quantified by recording the absorbance at 260 and 280 nm, and typically, 2 µL was used for PCR amplification of various gene targets [37].

All *Aspergillus* section *Fumigati*, *Aspergillus* section *Flavi*, and *Aspergillus* section *Terrei* isolates (*n* = 406) and some randomly selected isolates (*n* = 125) of other *Aspergillus* or mold species were identified by PCR amplification and/or PCR-sequencing of various gene targets. *Aspergillus* section *Fumigati*, *Aspergillus* section *Flavi*, and *Aspergillus* section *Terrei* isolates were identified by PCR using AFUF2 and AFUR2 primer pair, AFLF2 and AFLR2 primer pair, and ATEF2 and ATER2 primer pair (Supplementary Table S2), respectively, as described previously [10,37,38]. Other *Aspergillus* and mold species isolates were identified by PCR-sequencing of the internally transcribed spacer (ITS) region of ribosomal DNA (rDNA) and/or by partial sequencing of β-tubulin and calmodulin gene fragments by using ITS1 and ITS4 primer pair, BTUBF and BTUBR primer pair, and Cmd5 and Cmd6 primer pair (Supplementary Table S2), respectively, as described previously [39,40]. PCR amplicons of rDNA and β-tubulin and calmodulin gene fragments were sequenced with internal primers (Supplementary Table S2), as described in detail previously [39,40]. GenBank basic local alignment search tool (BLAST) searches (https://blast.ncbi.nlm.nih.gov/Blast.cgi, accessed on 28 June 2024) were performed for species identification, and sequence identity >99% was used to define con-specific strains.

### 2.3. Antifungal Drug Susceptibility Testing

Antifungal susceptibility of 117 clinical, 23 randomly selected environmental *A. fumigatus*, and nine rare *Aspergillus* spp. isolates to various antifungal drugs were carried out by Etest (AB Biodisk, bioMerieux, Marcy l’-Etoile, France) according to manufacturer’s instructions and as described previously [18,34,41]. Briefly, *A. fumigatus* isolates were freshly sub-cultured on the surface of potato dextrose agar (PDA) to obtain good sporulation; the spores were suspended in 0.9% saline and 0.2% Tween 80; the conidial suspension was evenly spread on Roswell Park Memorial Institute (RPMI) 1640 medium agar plates supplemented with 2% glucose (pH adjusted to 7.0 with morpholinepropanesulfonic acid buffer); and minimum inhibitory concentration (MIC) values were read after 24 h at 35 °C. Reference *A. fumigatus* strain (CBS 113.26) was included with each batch to ensure quality control. *A. fumigatus* isolates with MIC values of ≥2 µg/mL and ≥2 µg/mL were considered resistant to itraconazole and voriconazole, respectively [41,42,43]. Although clinical breakpoints for rare aspergilli are not available, MIC values of ≥2 µg/mL, ≥2 µg/mL, ≥2 µg/mL, ≥0.5 µg/mL, and ≥0.5 µg/mL were considered resistant to itraconazole, voriconazole posaconazole, anidulafungin and micafungin, respectively [41].

### 2.4. Ehrlich Test

The Ehrlich test for the detection of production of cyclopiazonic acid or related alkaloids by the environmental isolates of rare *Aspergillus* spp. was performed as described previously [44]. Briefly, the agar plug was cut out from the center of the colony of rare *Aspergillus* spp. isolate, and the filter paper soaked in Ehrlich reagent (2 g of 4-dimethylamino-benzaldehyde added to 85 mL of 96% ethanol and 15 mL of 10 N HCl) was placed on the mycelial side of the agar plug. The positive result indicating the presence of cyclopiazonic acid or related alkaloids was inferred if a violet ring appeared after 2–6 min of incubation at room temperature.

### 2.5. Detection of Mutations in the Promoter and Coding Regions of cyp51A

Resistance to triazoles in clinical *A. fumigatus* isolates usually involves single nucleotide mutations at some codon positions in the *cyp51A* gene. Resistance mechanisms involving tandem repeats (TR_34_ or TR_46_) in the promoter region together with nonsynonymous mutation(s) in *cyp51A* (TR_34_/L98H or TR_46_/Y121F/T289A) have also been detected in triazole-resistant *A. fumigatus* isolates recovered from various environmental sources, treatment naïve human subjects as well as from patients under treatment [25,26,27,28,33,34,35]. The presence of tandem repeats in the promoter region and nonsynonymous mutations in the structural *cyp51A* gene was detected by PCR amplification and/or by PCR-sequencing of three overlapping gene fragments by using three different primer pair combinations (Supplementary Table S2), as described previously [35,45]. The sequences of the assembled *cyp51* gene were compared with the corresponding wild-type sequence from reference *A. fumigatus* strain Af293 and *A. fumigatus* strain VPCI1042/09 containing the TR_34_/L98H mutations.

### 2.6. Short Tandem Repeat (STR) Typing

Molecular fingerprinting of clinical and environmental *A. fumigatus* isolates (*n* = 70) from Kuwait was performed by STR typing by using a panel of nine short tandem repeats, as described previously [46]. Briefly, three separate multiplex PCRs amplifying three dinucleotide repeat loci, three trinucleotide repeat loci, and three tetranucleotide repeat loci were carried out. The three forward primers in each multiplex PCR assay were labeled with carboxyfluorescein (FAM), hexachlorofluorescein (HEX), or tetrachlorofluorescein (TET) dyes at the 5′ end, respectively. Other reaction and cycling conditions were same as those described in detail previously [46]. The amplified fragments were loaded on an automated DNA sequencer, and the data were analyzed as described in detail previously [46]. Duplicate data were generated for 6 clinical *A. fumigatus* isolates to validate the microsatellite data. A dendrogram was generated by using STR data, treated as categorical values, by using BioNumerics v.7.6.1 (Applied Maths, Sint-Martens-Latem, Belgium) program with Unweighted Pair Group Method with Arithmetic Mean (UPGMA) cluster algorithm [46]. The microevolution among *A. fumigatus* isolates was also studied by using the minimal spanning tree algorithm of the BioNumerics (v.7.6.1 software) software, as described previously [46].

## 3. Results

During the study period of 17 months, a total of 6179 isolates of filamentous fungi were cultured from different indoor and outdoor locations. Of these, 1773 (29.6%) originated from indoor environments (two hospital wards and three ICUs) and 4406 (71.3%) from the outdoor environment (Table 1). Overall, 19 different genera of filamentous fungi were recorded. *Cladosporium* spp. (*n* = 2311, 37%), *Aspergillus* spp. (*n* = 1327, 22%), *Penicillium* spp. (*n* = 1325, 21%), *Paecelomyces* spp. (*n* = 473, 8%), and *Alternaria* spp. (*n* = 218, 4%) were the five most commonly isolated molds, both in outdoor air (*n* = 1792, *n* = 936, *n* = 834, *n* = 266 and *n* = 190, respectively) and the hospital indoor air/environment (*n* = 519, *n* = 391 and *n* = 491, *n* = 207 and *n* = 28, respectively) (Table 1). Of the remaining 525 isolates, 133 (2%) were identified as *Bipolaris* spp., while 199 (3%) isolates belonged to 13 other genera. A total of 193 (3%) isolates remained unidentified (Table 1). Among the six major fungi, the isolation frequency of *Cladosporium* spp. and *Aspergillus* spp. was nearly same at ground level and 15 m above ground level. However, the isolation frequency of *Penicillium* spp. and *Paecelomyces* spp. was higher at ground level compared to 15 m above ground level, while the isolation frequency of *Alternaria* spp. and *Bipolaris* spp. was lower at ground level compared to 15 m above ground level.

Of 1327 *Aspergillus* spp. isolates, 862 (65%) were identified as *A. niger*, 182 (13.7%) as *A. flavus*, 166 (12.5%) as *A. fumigatus*, 58 (4.4%) as *A. terreus*, 31 (2.3%) as *A. nidulans*, 7 (0.5%) as *A. versicolor*, 7 (0.5%) as *A. calidoustus*, and 14 (1.1%) isolates as other *Aspergillus* spp. (Table 2). Among *Aspergillus* spp., the relative prevalence of *A. fumigatus* in indoor environments was 9.9%, whereas in outdoor environments, it was 18% at ground level and 9.6% at 15 m above ground level. Among non-*A. fumigatus* spp. (barring *A. niger*), *A flavus* showed a greater prevalence than *A. terreus* in both indoor (13.2% vs. 5.8%) and outdoor (15% vs. 3.2%) environments. The overall prevalence of members of the sections *Fumigati*, *Flavi*, and *Terrei* among total indoor molds was 6.4% compared to 7.6% among outdoor molds. In order to determine the temporal trend in the occurrence of molds in the outdoor environment, the occurrence of *Aspergillus* spp. and other molds during a one-year period was calculated, and the data are presented in Table 3. The data showed two apparent peaks for the prevalence of molds in outdoor environments: a major peak during October–November and a minor peak during April–May. Soil samples were also screened at multiple time points and yielded 13 additional *A. fumigatus* isolates that are not included in Table 1.

Nine of the fourteen unidentified *Aspergillus* spp. isolates were available (the remaining five cultures could not be revived) for species-specific identification and in vitro antifungal susceptibility testing, and the results are presented in Table 4. The rDNA sequencing identified these nine isolates as *A. flavipes*, *A. ochraceus*, *A. hiratsukae*, *A. intermedium*, *A. amstelodami*, and *A. insuetus* from hospital environments and *A. ustus*, *A. chivalieri*, and *A. muricatus* from outdoor environments (Table 4). The Ehrlich test for the detection of cyclopiazonic acid or related alkaloids was negative for all nine isolates. Growth at higher temperatures (42 °C) showed that only *A. chevalieri* and *A. muricatus* were able to grow while the remaining isolates showed no growth. Susceptibility testing showed that all nine isolates appeared to be susceptible to the three triazoles and the two echinocandins tested (Table 4).

Susceptibility testing was also carried out for 117 clinical *A. fumigatus* isolates for itraconazole and voriconazole, and the results are presented in Table 5. Two isolates were resistant to itraconazole and voriconazole. The remaining 115 isolates were susceptible to both triazoles. As expected, 15 and 8 environmental *A. fumigatus* isolates from two previous studies [33,34] were again detected as susceptible and resistant to both itraconazole and voriconazole, respectively.

The PCR-sequencing of the *cyp51A* gene identified TR_34_/L98H mutations in two clinical (Kw1431/10-c3 and Kw2772/11-c6) isolates recovered from sputum samples of patients in Hospital A, one isolate from outdoor air (E218) outside Hospital A, three (E076, E119 and E454) isolates from the environment (air or floor) of Hospital A, and four isolates from soil samples (two isolates, R015 and R018 from Shuwaikh area and two isolates, R043-c8 and R044 from Jaleeb Al-Shuyoukh area with both locations being 12–15 km away from Hospital A).

**Figure 1:**
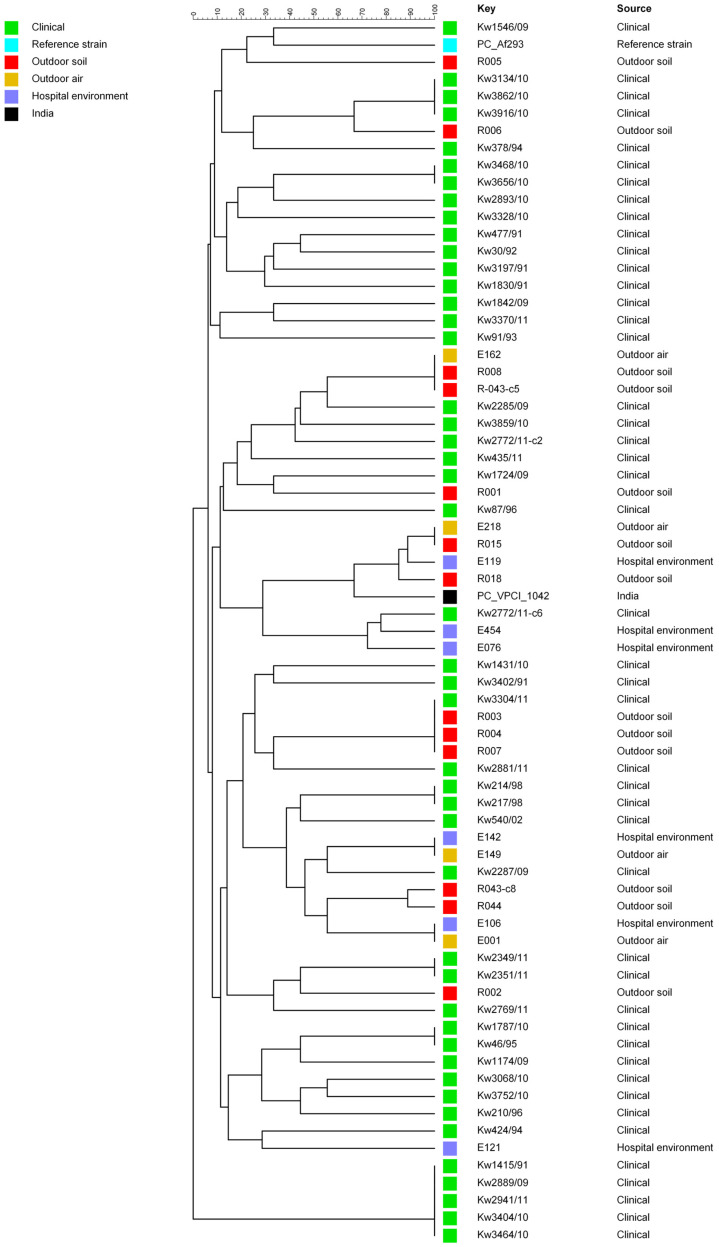
Genotypic relationship among environmental and clinical *A. fumigatus* isolates from Kuwait. The dendrogram is based on a categorical analysis of nine microsatellite markers in combination with UPGMA clustering. The scale bar represents the percentage identity.

Molecular fingerprinting by STR typing was carried out for 70 *A. fumigatus* isolates. These included 47 clinical isolates, 13 isolates from outdoor soil samples from different parts of Kuwait, 6 isolates from the environment (air or floor) of Hospital A, and 4 isolates from outdoor air (outside Hospital A). These isolates comprised 60 itraconazole-susceptible clinical (*n* = 45) and environmental (*n* = 15) isolates and 10 itraconazole-resistant *A. fumigatus*. The itraconazole-resistant isolates included two clinical isolates (Kw1431/10-c3 and Kw2772/11-c6) from Hospital A, one isolate (E218) from outdoor air outside Hospital A, three isolates (E076, E119 and E454) from indoor air/environment of Hospital A, and four (R015, R018, R043-c8 and R044) isolates from soil samples. Additionally, duplicate DNA samples prepared independently from six clinical isolates were also included. Two reference strains, PC-Af293 (representing triazole-susceptible *A. fumigatus*) and PC_VPCI-1042 (representing triazole-resistant *A. fumigatus* containing TR_34_/L98H mutations in *cyp51A*), were also included. The STR data showed that 41 isolates yielded a unique pattern, while 31 isolates clustered in 12 MST patterns, including 5 (Kw1415/91, Kw2889/09, Kw3404/10, Kw3464/10, and Kw2941/11) isolates that clustered due to lack of STR loci (Figure 1). Duplicate DNA samples from six isolates yielded identical results confirming the accuracy of STR typing.

Among clustered isolates, four clusters included clinical isolates recovered during the same year, while two clusters included two clinical isolates each recovered in different years. More importantly, two clusters contained *A. fumigatus* isolates (E142 + E149 and E001 and E106) recovered from outdoor air and the indoor environment of Hospital A, another cluster contained three (E162, R008, and R043-c6) isolates recovered from outdoor air/soil samples from areas close to Hospital A, and another cluster contained four (R003, R004, R007 and Kw3304/11) isolates recovered from outdoor soil samples (R003, R004, and R007) from areas close to Hospital A and from a clinical specimen (Kw3304/11) of a patient from Hospital A (Figure 1). Another cluster included two isolates recovered from air outside Hospital A and soil samples from another location within Kuwait and both these isolates were also resistant to itraconazole. Furthermore, two of ten triazole-resistant isolates were closely related and were isolated from one locality (Jaleeb Al-Shoyoukh), while the remaining eight triazole-resistant isolates, including the two clinical isolates, Kw1431/10-c3 and Kw2772/11-c6, formed a distinct group, which also included the reference triazole-resistant *A. fumigatus* isolate (PC-VPCI_1042) from India used in this study (Figure 1).

**Figure 2:**
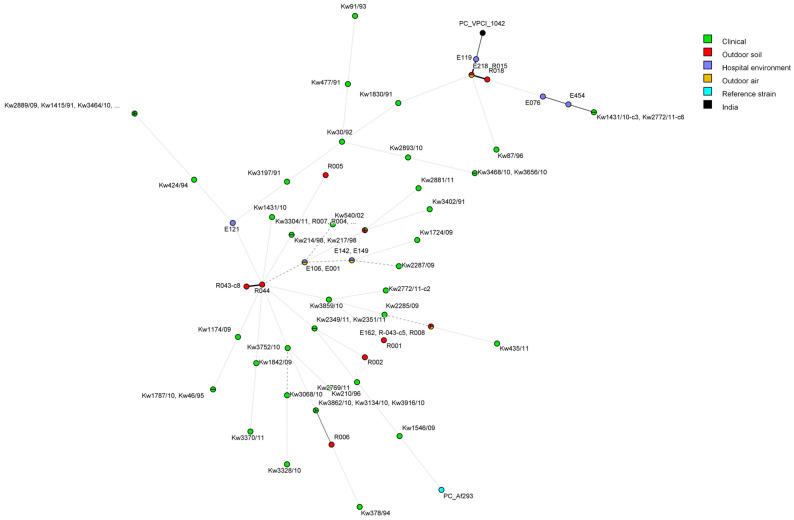
Minimum spanning tree showing genotypic relationship among clinical and environmental *A. fumigatus* isolates in Kuwait. Each circle corresponds to a unique genotype, and lines between circles represent relative distance between isolates. Connecting lines correspond to the number of microsatellite loci differences between genotypes, with a solid thick line connecting genotypes that differ in one locus, a solid thin line connecting genotypes that differ in two to three loci, a dashed line connecting genotypes that differ in four loci, and a dotted line connecting genotypes that differ in more than four loci.

The microevolution among *A. fumigatus* isolates was also studied by using the minimal spanning tree algorithm of the BioNumerics (v.7.6.1 software) software, and the data are presented in Figure 2. These data also showed, on one hand, great diversity among clinical and environmental *A. fumigatus* isolates in Kuwait and, on the other hand, also confirmed close relationship among eight of 10 itraconazole-resistant isolates (including two clinical isolates and six isolates from indoor/outdoor hospital environment) among each other, as well as with the triazole-resistant isolate (PC_VPCI-1042) from India used as a reference strain.

## 4. Discussion

The total area of the State of Kuwait in the Arabian Peninsula mostly comprises desert or desert-like landscape, and the country is regarded as having the world’s highest particulate matter concentration in the outdoor air due to frequent dust storms occurring throughout the year [47]. Similar conditions also exist in other nearby countries like Saudi Arabia, Qatar, and the United Arab Emirates. It is, therefore, not surprising that the three countries of the Arabian Peninsula including Kuwait were found to have high observed severe exacerbation rates (defined as events requiring oral corticosteroids for ≥3 days or asthma-related emergency department visit or hospitalization) of asthma in a study involving adult patients from 17 different countries spread on five continents that looked at variations in severe exacerbation rates [48]. A higher risk of all-cause mortality has also been noted due to poor air quality and particulate pollution during days of low visibility/dust storms in another study from Kuwait [49].

During the screening of environmental samples in and around a major hospital in Kuwait for a continuous period of 17 months, 1773 and 4406 CFUs of filamentous fungi were isolated from different indoor and outdoor samples, respectively. Although 19 different genera of filamentous fungi were recovered, the five most commonly isolated organisms from both indoor and outdoor locations (comprising 92% of all CFUs) included *Cladosporium* spp. (37%), *Aspergillus* spp. (22%), *Penicillium* spp. (21%), *Paecelomyces* spp. (8%), and *Alternaria* spp. (4%), and their isolation was higher from outdoor air than an indoor environment. These organisms, particularly *Cladosporium* spp., are also among the most commonly isolated fungi from indoor/outdoor samples, and their outdoor concentrations are generally higher than those indoors [50,51]. They are also commonly found as the dominant molds in other nearby countries with similar environmental (dry, arid, and dust-laden) conditions such as Qatar, Saudi Arabia, UAE, and Iran [52,53,54,55,56]. The exposure of vulnerable individuals to spores of *Cladosporium* spp., *Penicillium* spp., and *Alternaria* spp. is known to be associated with reduced lung function and increased airway inflammation, while exposure to *Aspergillus* spp. spores is a well-known risk factor for the development of allergic bronchopulmonary aspergillosis [8,12,57,58]. Previous sensitization with *Aspergillus* antigens has also been detected in 16% to 38% of asthmatic subjects in different geographical regions [15]. The majority (862 of 1327, 65%) of aspergilli belonged to the *A. niger* species complex in Kuwait. *Aspergillus niger* species complex members were also detected as the most common aspergilli in the outdoor air/house dust of Riyadh, Saudi Arabia, and Tehran, Iran [59,60,61].

Temporal trends showed that the isolation frequency of molds including *Aspergillus* spp. was high during the months of October to December and then declined, but a minor peak was also apparent during the months of April–May. These data coincide with the peak allergy season of October–November and a minor peak in April–May, which were recorded in Kuwait [62,63].

A few rare aspergilli were also detected and identified by rDNA sequencing from the indoor environment (including ICUs) of Hospital A, including *A. flavipes*, *A. ochraceus*, *A. hiratsukae*, *A. intermedium*, *A. amstelodami*, and *A. insuetus*, while *A. ustus*, *A. chevalieri*, and *A. muricatus* were isolated from the outdoor air. It is pertinent to mention here that *A. flavipes*, *A. ochraceus*, and *A. ustus* have also been isolated from outdoor air or house dust in Riyadh, Saudi Arabia [59,60,61]. *A. ochraceus*, *A. hiratsukae*, *A. intermedium*, and *A. insuetus* have previously been isolated from clinical specimens [13,64]. Although these rare aspergilli were susceptible to the antifungal drugs, the isolation of *A. ochraceus*, *A. hiratsukae*, *A. intermedium*, and *A. insuetus* from the indoor hospital environment is worrisome, as, in addition to ABPA, invasive infections by some of these aspergilli have been reported among immunocompetent, hospitalized patients [65,66,67]. Only *A. chevalieri* and *A. muricatus* showed detectable growth at 42 °C and grew from outdoor air. While *A. muricatus* is known to produce ochratoxin A and is likely non-pathogenic for humans, *A. chevalieri* is known to cause cutaneous aspergillosis as well as invasive pulmonary aspergillosis in susceptible patients [68,69].

Molecular fingerprinting done by STR analyses for 70 clinical and environmental *A. fumigatus* isolates revealed several interesting findings. These included the clustering of isolates with identical genotypes from outdoor air and the indoor environment of Hospital A (E001 and E106 as well as E142 and E149) or isolates recovered from outdoor soil samples (R003, R004, and R007) from areas close to Hospital A and from a clinical specimen (Kw3304/11) of a patient from Hospital A. These findings suggest that the patient from Hospital A was likely infected with an environmental *A. fumigatus* isolate prevalent in the area, as has been reported previously in other studies involving both triazole-susceptible and triazole-resistant *A. fumigatus* isolates [70,71,72]. Of the eight triazole-resistant isolates carrying TR_34_/L98H mutations in *cyp51A*, one triazole-resistant isolate (E218) air sample from outside Hospital A was identical (R015) or closely related (R018) to two triazole-resistant isolates from soil samples from the Shuwaikh area. Interestingly, three triazole-resistant (E076, E119, and E454) isolates from the environment of Hospital A and the two triazole-resistant clinical (Kw1431/10-c3 and Kw2772/11-c6) isolates from patients of Hospital A formed a distinct group that also included the three (E218, R015, and R018) isolates mentioned above and the reference triazole-resistant *A. fumigatus* isolate (PC-VPCI_1042) from India. The minimal spanning tree also confirmed a close relationship among eight of ten itraconazole-resistant isolates (including two clinical isolates and six isolates from indoor/outdoor hospital environment), as well as with the triazole-resistant isolate (PC_VPCI-1042) from India. These findings demonstrate a possible environmental route of infection with triazole-resistant strains among patients of Hospital A, and their close relationship with the triazole-resistant control isolate as has also been demonstrated in a few other studies [73,74,75].

Surprisingly, two other triazole-resistant isolates from soil samples from another locality (Jaleeb Al-Shuyoukh) were genetically different. It is pertinent to mention here that the soil and manure used in Kuwait for gardening/agricultural purposes are procured from several Asian and European countries, and it is probable that the two clinical and the majority of environmental itraconazole-resistant isolates in Kuwait were introduced from India, while the remaining two soil isolates originated from elsewhere. The introduction of azole-resistant *Aspergillus* across national borders via agricultural products has been reported previously [76,77]. The presence of triazole-resistant *A. fumigatus* isolates in the environment in Kuwait and their isolation from patients of Hospital A is a worrisome development, as invasive aspergillosis among immunocompromised patients caused by triazole-resistant *A. fumigatus* is associated with higher mortality compared to patients infected with triazole-susceptible isolates [25,78]. Recent guidelines from the European Society of Clinical Microbiology and Infectious Diseases-European Confederation of Medical Mycology-European Respiratory Society (ESCMID-ECMM-ERS) recommend resistance testing of all significant clinical *A. fumigatus* isolates and performing regular resistance surveillance program [25,79].

## 5. Conclusions

A total of 6179 colonies of filamentous fungi, mainly including *Cladosporium* spp., *Aspergillus* spp., *Penicillium* spp., *Paecilomyces* spp., and *Alternaria* spp., were grown during a 17-month screening of indoor and outdoor environmental samples of Hospital A in Kuwait. These environmental molds and aspergilli are an important source of opportunistic infections and seasonal allergies in susceptible patients. The isolation frequency was higher during October/November, a minor peak was apparent during April–May, and both these periods coincide with the peak allergy seasons in Kuwait. A few other rare aspergilli with pathogenic potential were also obtained from the indoor hospital environment. Itraconazole-resistant *A. fumigatus* with mutations TR_34_/L98H in *Cyp51A* were recovered from outdoor air, the hospital environment, and clinical samples. Molecular STR fingerprinting showed that eight triazole-resistant environmental and clinical isolates carrying the TR_34_/L98H mutations had a close genetic relationship, forming a distinct group, while the other two triazole-resistant isolates from soil samples were genetically different. These findings suggest an environmental route of infection with triazole-resistant strains among some patients.

## Figures and Tables

**Table 1:** Species distribution of fungi based on colony characteristics of molds obtained from sampling of outdoor air and indoor hospital air and other environments.

Molds Isolated	Number of Colony-Forming Units (CFUs) Recovered from							Total
	Indoor Hospital Samples					Outdoor Air		
	MICU	I-ICU	SICU	Ward-3	Ward-5	Ground Level	Above Ground Level *	
*Cladosporium* spp.	27	21	21	261	189	912	880	**2311**
*Aspergillus* spp.	59	64	68	105	95	430	506	**1327**
*Penicillium* spp.	128	75	61	119	108	312	522	**1325**
*Pacelomyces* spp.	48	15	27	40	77	64	202	**473**
*Alternaria* spp.	0	2	1	10	15	118	72	**218**
*Bipolaris* spp.	1	0	1	7	2	70	52	**133**
*Neurospora crassa*	0	4	1	0	0	16	46	**67**
*Rhizopus* spp.	4	0	0	7	0	22	4	**37**
*Fusarium* spp.	0	1	0	9	7	2	12	**31**
*Nigrospora sphaerica*	0	0	0	0	0	20	2	**22**
*Ulocladium* spp.	0	0	0	0	0	18	4	**22**
*Syncephalastrum* spp.	0	1	0	2	2	0	0	**5**
*Chaetomium* spp.	0	0	0	2	3	0	0	**5**
*Acremonium* spp.	0	0	0	0	0	2	0	**2**
*Mucor circinelloides*	0	0	0	0	0	0	2	**2**
*Scopulariopsis* spp.	0	0	0	0	0	0	2	**2**
*Chrysosporium* spp.	0	0	0	0	0	0	2	**2**
*Curvularia* spp.	2	0	0	0	0	0	0	**2**
Unidentified	6	16	7	28	24	56	56	**193**
**Total Molds**	**275**	**199**	**187**	**590**	**522**	**2042**	**2364**	**6179**

MICU—Medical intensive care unit, SICU—Surgical ICU, I-ICU—Isolation ICU. *Aspergillus* species represent their respective sections. * Air sampling was done at 15 m above ground level.

**Table 2:** Distribution of *Aspergillus* spp. obtained during sampling of outdoor air and indoor hospital air and environment.

*Aspergillus* Species	Number of Colony-Forming Units (CFUs) Recovered from							Total
	Indoor Hospital Samples					Outdoor Air		
	MICU	I-ICU	SICU	Ward-3	Ward-5	Ground Level	Above Ground Level *	
*A. fumigatus*	9	6	5	8	11	78	49	**166**
*A. terreus*	3	4	9	4	3	14	21	**58**
*A. flavus*	4	15	8	9	16	65	65	**182**
*A. niger*	35	37	34	76	62	256	362	**862**
*A. nidulans*	2	1	3	4	2	11	8	**31**
*A. versicolor*	1	0	0	3	1	2	0	**7**
*A. calidoustus*	3	0	3	0	0	1	0	**7**
Other *Aspergillus* spp.	2	1	6	1	0	3	1	**14**
**Total**	**59**	**64**	**68**	**105**	**95**	**430**	**506**	**1327**

MICU—Medical intensive care unit, SICU—Surgical ICU, I-ICU—Isolation ICU. *Aspergillus* species represent their respective sections. * Air sampling was done at 15 m above ground level.

**Table 3:** Distribution of *Aspergillus* spp. and other non-*Aspergillus* molds obtained during sampling of outdoor air and indoor hospital air and environment during a one-year period.

Month	No. of Isolates of		Total Molds Isolated
	*Aspergillus* spp.	Non-*Aspergillus* Molds	
October	230	1110	**1340**
November	110	446	**556**
December	170	232	**402**
January	92	146	**238**
February	32	338	**370**
March	42	158	**200**
April	38	188	**226**
May	60	218	**278**
June	70	110	**180**
July	48	74	**122**
August	2	238	**240**
September	42	212	**254**
**Total**	**936**	**3470**	**4406**

**Table 4:** Uncommon *Aspergillus* spp. isolated from indoor/outdoor air and their susceptibility to antifungal drugs.

*Aspergillus* Species	Source of Isolation	Colony Diameter (in mm) Grown at			Minimum Inhibitory Concentration (MIC) (µg/mL) for				
		30 °C	37 °C	42 °C	ITR	VOR	POS	ANF	MFG
*A. flavipes*	Medical ICU	16	17	NG	0.75	0.19	0.5	0	0.003
*A. ochraceus*	Medical ICU	30	32	NG	0.25	0.047	0.25	0.01	0.5
*A. hiratsukae*	Isolation ICU	12	18	NG	0.25	0.05	0.25	0.01	0.5
*A. intermedium*	Surgical ICU	7	6	NG	0.5	0.01	0.125	0.003	0.003
*A. amstelodami*	Surgical ICU	15	18	NG	0.5	1.5	0.38	0.004	0.047
*A. insuetus*	Ward 3	31	28	NG	0.5	0.19	0.25	0.004	0.003
*A. ustus*	Outdoo air	45	40	NG	0.5	0.008	0.125	0.003	0.003
*A. chevalieri*	Outdoor air	65	80	15	0.016	0.75	0.016	0	0.002
*A. muricatus*	Outdoor air *	35	56	17	0.064	0.094	0.047	0.008	0.006

ITR, itraconazole; VOR, voriconazole; POS, posaconazole; ANF, anidulafungin; MFG, micafungin; NG, no growth. * Outdoor air sampling at 15 m above ground level.

**Table 5:** In vitro antifungal susceptibility testing results of *A. fumigatus* isolates (*n* = 117) against itraconazole, voriconazole, and amphotericin B.

Antifungal Drug	No. of Isolates with Minimum Inhibitory Concentration (MIC) (µg/mL) of																		
	≤0.01	0.02	0.03	0.05	0.06	0.09	0.13	0.19	0.25	0.38	0.5	0.75	1	1.5	2	4	8	16	32
ITR	2		1	2		1	4	8	5	10	17	32	18	15			**1** *		**1** *
VOR	2	1	7	13	4	17	24	25	13	5	4				**1** *		**1** *		

ITR, itraconazole; VOR, voriconazole. The MIC values indicative of resistance are shown in bold face numbers with an asterisk (*) and modal MIC values are underlined.

## Data Availability

The original contributions presented in the study are included in the article/Supplementary Materials, further inquiries can be directed to the corresponding authors.

## Supplementary Materials

The following supporting information can be downloaded at: https://www.mdpi.com/article/10.3390/jof11020083/s1, Table S1: No. and source of clinical *A. fumigatus* isolates used in this study; Table S2: Nucleotide sequences and specific purpose of primers used in PCR-amplification or DNA sequencing of various genomic regions of *Aspergillus* spp. isolates and the expected sizes of amplicons, where applicable, in base pairs (bp).

## References

[1] Brown G.D., Denning D.W., Gow N.A., Levitz S.M., Netea M.G., White T.C. (2012). Hidden killers: Human fungal infections. Sci. Transl. Med..

[2] Ahmad S., Joseph L., Parker J.E., Asadzadeh M., Kelly S.L., Meis J.F., Khan Z. (2019). *ERG6* and *ERG2* are major targets conferring reduced susceptibility to amphotericin B in clinical *Candida glabrata* isolates in Kuwait. Antimicrob. Agents Chemother..

[3] Wiederhold N.P. (2021). Emerging fungal infections: New species, new names, and antifungal resistance *Clin*. Chem..

[4] Ahmad S., Asadzadeh M. (2023). Strategies to prevent the transmission of *Candida auris* in health care facilities. Curr. Fung. Infect. Rep..

[5] Zhang Z., Bills G.F., An Z. (2023). Advances in the treatment of invasive fungal disease. PLoS Pathog..

[6] Ahmad S., Asadzadeh M., Al-Sweih N., Khan Z. (2024). Spectrum and management of rare *Candida*/yeast infections in Kuwait in the Middle East. Therap. Adv. Infect. Dis..

[7] de S Araújo G.R., Souza W., Frases S. (2017). The hidden pathogenic potential of environmental fungi. Future Microbiol..

[8] Denham S.T., Wambaugh M.A., Brown J.C.S. (2019). How environmental fungi cause a range of clinical outcomes in susceptible hosts. J. Mol. Biol..

[9] Machiavello Roman F.J., Pischel L., Azar M.M. (2024). Lung infections due to emerging fungal pathogens. Curr. Opin. Pulm. Med..

[10] Khan Z.U., Ahmad S., Mokaddas E., Said T., Nair M.P., Halim M.A., Nampoory M.R., McGinnis M.R. (2007). Cerebral aspergillosis diagnosed by detection of *Aspergillus flavus*-specific DNA, galactomannan and (1-->3)-β-D-glucan in clinical specimens. J. Med. Microbiol..

[11] Mokaddas E., Burhamah M.H., Ahmad S., Khan Z.U. (2010). Invasive pulmonary aspergillosis due to *Aspergillus terreus*: Value of DNA, galactomannan, and (1–3)-β-D-glucan detection in serum samples as adjunct to diagnosis. J. Med. Microbiol..

[12] Kosmidis C., Denning D.W. (2015). The clinical spectrum of pulmonary aspergillosis. Thorax.

[13] Lucio J., Alcazar-Fuoli L., Gil H., Cano-Pascual S., Hernandez-Egido S., Cuetara M.S., Mellado E. (2024). Distribution of *Aspergillus* species and prevalence of azole resistance in clinical and environmental samples from a Spanish hospital during a three-year study period. Mycoses.

[14] Evans T.J., Lawal A., Kosmidis C., Denning D.W. (2024). Chronic pulmonary aspergillosis: Clinical presentation and management. Semin. Respir. Crit. Care Med..

[15] Shah A., Panjabi C. (2014). Allergic aspergillosis of the respiratory tract. Eur. Respir. Rev..

[16] Kontoyiannis D.P., Marr K.A., Park B.J., Alexander B.D., Anaissie E.J., Walsh T.J., Ito J., Andes D.R., Baddley J.W., Brown J.M. (2010). Prospective surveillance for invasive fungal infections in hematopoietic stem cell transplant recipients, 2001–2006: Overview of the Transplant-Associated Infection Surveillance Network (TRANSNET) Database. Clin. Infect. Dis..

[17] Dimopoulos G., Frantzeskaki F., Poulakou G., Armaganidis A. (2012). Invasive aspergillosis in the intensive care unit. Ann. N. Y. Acad. Sci..

[18] Al-Wathiqi F., Ahmad S., Khan Z. (2013). Molecular characterization and antifungal susceptibility profile of *Aspergillus flavus* isolates recovered from clinical specimens in Kuwait. BMC Infect. Dis..

[19] Wang Y., Zhang L., Zhou L., Zhang M., Xu Y. (2022). Epidemiology, drug susceptibility, and clinical risk factors in patients with invasive aspergillosis. Front. Public Health.

[20] Kanaujia R., Singh S., Rudramurthy S.M. (2023). Aspergillosis: An update on clinical spectrum, diagnostic schemes, and management. Curr. Fungal Infect. Rep..

[21] Koulenti D., Papathanakos G., Blot S. (2023). Invasive pulmonary aspergillosis in the ICU: Tale of a broadening risk profile. Curr. Opin. Crit. Care..

[22] Machado M., Fortún J., Muñoz P. (2024). Invasive aspergillosis: A comprehensive review. Med. Clin..

[23] Feys S., Carvalho A., Clancy C.J., Gangneux J.P., Hoenigl M., Lagrou K., Rijnders B.J.A., Seldeslachts L., Vanderbeke L., van de Veerdonk F.L. (2024). Influenza-associated and COVID-19-associated pulmonary aspergillosis in critically ill patients. Lancet Respir. Med..

[24] Cadena J., Thompson G.R., Patterson T.F. (2021). Aspergillosis: Epidemiology, diagnosis, and treatment. Infect. Dis. Clin. N. Am..

[25] Lestrade P.P.A., Meis J.F., Melchers W.J.G., Verweij P.E. (2019). Triazole resistance in *Aspergillus fumigatus*: Recent insights and challenges for patient management. Clin. Microbiol. Infect..

[26] Wiederhold N.P., Verweij P.E. (2020). *Aspergillus fumigatus* and pan-azole resistance: Who should be concerned?. Curr. Opin. Infect. Dis..

[27] Allizond V., Comini S., Bianco G., Costa C., Boattini M., Mandras N., Banche G., Cuffini A.M., Cavallo R. (2021). Exposure to the agricultural fungicide tebuconazole promotes *Aspergillus fumigatus* cross-resistance to clinical azoles. New Microbiol..

[28] Verweij P.E., Song Y., Buil J.B., Zhang J., Melchers W.J.G. (2024). Antifungal resistance in pulmonary aspergillosis. Semin. Respir. Crit. Care Med..

[29] Sewell T.R., Zhang Y., Brackin A.P., Shelton J.M.G., Rhodes J., Fisher M.C. (2019). Elevated prevalence of azole-resistant *Aspergillus fumigatus* in urban versus rural environments in the United Kingdom. Antimicrob. Agents Chemother..

[30] Bosetti D., Neofytos D. (2023). Invasive aspergillosis and the impact of azole-resistance. Curr. Fungal Infect. Rep..

[31] Loukou E., Jensen N.F., Rohde L., Andersen B. (2024). Damp buildings: Associated fungi and how to find them. J. Fungi.

[32] Khan Z., Ahmad S., Joseph L. (2014). Aerial prevalence of *Aspergillus calidoustus* isolates in and around a tertiary care hospital in Kuwait and assessment of their pathogenicity. J. Clin. Microbiol..

[33] Ahmad S., Khan Z., Hagen F., Meis J.F. (2014). Occurrence of triazole-resistant *Aspergillus fumigatus* with TR_34_/L98H mutations in outdoor and hospital environment in Kuwait. Environ. Res..

[34] Ahmad S., Joseph L., Hagen F., Meis J.F., Khan Z. (2015). Concomitant occurrence of itraconazole-resistant and -susceptible strains of *Aspergillus fumigatus* in routine cultures. J. Antimicrob. Chemother..

[35] Asadzadeh M., Alobaid K., Ahmad S., Mazloum S. (2023). First report of azole-resistant *Aspergillus fumigatu* with TR46/Y121F/T289A mutations in Kuwait and an update on their occurrence in the Middle East. J. Fungi.

[36] Hageskal G., Knutsen A.K., Gaustad P., de Hoog G.S., Skaar I. (2006). Diversity and significance of mold species in Norwegian drinking water. Appl. Environ. Microbiol..

[37] Khan Z.U., Ahmad S., Theyyathel A.M. (2008). Detection of *Aspergillus fumigatus*-specific DNA, (1–3)-β -D-glucan and galactomannan in serum and bronchoalveolar lavage specimens of experimentally infected rats. Mycoses.

[38] Ahmad S., Khan Z.U., Theyyathel A.M. (2007). Diagnostic value of DNA, (1–3)-β-D-glucan, and galactomannan detection in serum and bronchoalveolar lavage of mice experimentally infected with *Aspergillus terreus*. Diagn. Microbiol. Infect. Dis..

[39] Khan Z.U., Ahmad S., Hagen F., Fell J.W., Kowshik T., Chandy R., Boekhout T. (2010). *Cryptococcus randhawai* sp. nov., a novel anamorphic basidiomycetous yeast isolated from tree trunk hollow of *Ficus religiosa* (peepal tree) from New Delhi, India. Antonie Van Leeuwenhoek.

[40] Khan Z., Ahmad S., Al-Ghimlas F., Al-Mutairi S., Joseph L., Chandy R., Sutton D.A., Guarro J. (2012). *Purpureocillium lilacinum* as a cause of cavitary pulmonary disease: A new clinical presentation and observations on atypical morphologic characteristics of the isolate. J. Clin. Microbiol..

[41] Lass-Flörl C. (2013). Susceptibility testing in *Aspergillus* species complex. Clin. Microbiol. Infect..

[42] Burgel P.R., Baixench M.T., Amsellem M., Audureau E., Chapron J., Kanaan R., Honoré I., Dupouy-Camet J., Dusser D., Klaassen C.H. (2012). High prevalence of azole-resistant *Aspergillus fumigatus* in adults with cystic fibrosis exposed to itraconazole. Antimicrob. Agents Chemother..

[43] Guinea J. (2020). Updated EUCAST clinical breakpoints against *Aspergillus*, implications for the clinical microbiology laboratory. J. Fungi.

[44] Samson R.A., Noonim P., Meijer M., Houbraken J., Frisvad J.C., Varga J. (2007). Diagnostic tools to identify black aspergilli. Stud. Mycol..

[45] Ahmad S., Khan Z., Hagen F., Meis J.F. (2014). Simple, low-cost molecular assays for TR_34_/L98H mutations in the *cyp51A* gene for rapid detection of triazole-resistant *Aspergillus fumigatus* isolates. J. Clin. Microbiol..

[46] de Valk H.A., Meis J.F., Curfs I.M., Muehlethaler K., Mouton J.W., Klaassen C.H. (2005). Use of a novel panel of nine short tandem repeats for exact and high-resolution fingerprinting of *Aspergillus fumigatus* isolates. J. Clin. Microbiol..

[47] Al Salameen F., Habibi N., Uddin S., Al Mataqi K., Kumar V., Al Doaij B., Al Amad S., Al Ali E., Shirshikhar F. (2020). Spatio-temporal variations in bacterial and fungal community associated with dust aerosol in Kuwait. PLoS ONE.

[48] Lee T.Y., Price D., Yadav C.P., Roy R., Lim L.H.M., Wang E., Wechsler M.E., Jackson D.J., Busby J., Heaney L.G. (2024). International variation in severe exacerbation rates in patients with severe asthma. Chest.

[49] Achilleos S., Al-Ozairi E., Alahmad B., Garshick E., Neophytou A.M., Bouhamra W., Yassin M.F., Koutrakis P. (2019). Acute effects of air pollution on mortality: A 17-year analysis in Kuwait. Environ. Int..

[50] Haas D., Habib J., Luxner J., Galler H., Zarfel G., Schlacher R., Friedl H., Reinthaler F.F. (2014). Comparison of background levels of culturable fungal spore concentrations in indoor and outdoor air in southeastern Austria. Atm. Environ..

[51] Tseng C.C., Huang N., Hsieh C.J., Hung C.C., Guo Y.L. (2021). Contribution of visible surface mold to airborne fungal concentration as assessed by digital image quantification. Pathogens.

[52] Hasnain S.M., Al-Frayh A.S., Al-Suwaine A., Gad-El-Rab M.O., Fatima K., Al-Sedairy S. (2004). *Cladosporium* and respiratory allergy: Diagnostic implications in Saudi Arabia. Mycopathologia.

[53] Hasnain S.M., Al-Frayh A.S., Subiza J.L., Fernández-Caldas E., Casanovas M., Geith T., Gad-El-Rab M.O., Koshak E., Al-Mehdar H., Al-Sowaidi S. (2012). Sensitization to indigenous pollen and molds and other outdoor and indoor allergens in allergic patients from Saudi Arabia, United Arab Emirates, and Sudan. World Allergy Organ. J..

[54] Ziaee A., Zia M., Goli M. (2018). Identification of saprophytic and allergenic fungi in indoor and outdoor environments. Environ. Monit. Assess..

[55] Ghazanfari M., Yazdani Charati J., Keikha N., Kholoujini M., Kermani F., Nasirzadeh Y., Roohi B., Minooeianhaghighi M.H., Salari B., Jeddi S.A. (2022). Indoor environment assessment of special wards of educational hospitals for the detection of fungal contamination sources: A multi-center study (2019–2021). Curr. Med. Mycol..

[56] Yousefzadeh A., Maleki A., Athar S.D., Darvishi E., Ahmadi M., Mohammadi E., Tang V.T., Kalmarzi R.N., Kashefi H. (2022). Evaluation of bio-aerosols type, density, and modeling of dispersion in inside and outside of different wards of educational hospital. Environ. Sci. Pollut. Res. Int..

[57] Tham R., Erbas B., Dharmage S.C., Tang M.L., Aldakheel F., Lodge C.J., Thomas P.S., Taylor P.E., Abramson M.J., Lowe A.J. (2019). Outdoor fungal spores and acute respiratory effects in vulnerable individuals. Environ. Res..

[58] Agarwal R., Sehgal I.S., Muthu V., Denning D.W., Chakrabarti A., Soundappan K., Garg M., Rudramurthy S.M., Dhooria S., Armstrong-James D. (2024). Revised ISHAM-ABPA working group clinical practice guidelines for diagnosing, classifying and treating allergic bronchopulmonary aspergillosis/mycoses. Eur. Respir. J..

[59] Bahkali A.H., Paez S. (1999). Fungal flora in house dust in Riyadh, Saudi Arabia. Mycoses.

[60] El-Samawaty A.M.A., Yassin M.A., Abdel-Wahab M.A. (2018). Airborne fungi in outdoor air of Riyadh City, Kingdom of Saudi Arabia. Fresenius Environ. Bull..

[61] Aghaei-Gharehbolagh S., Shams-Ghahfarokhi M., Amanloo S., Razzaghi-Abyaneh M. (2018). Molecular characterization of aspergilli isolated from outdoor air. J. Mycol. Med..

[62] Behbehani N., Arifhodzic N., Al-Mousawi M., Marafie S., Ashkanani L., Moussa M., Al-Duwaisan A. (2004). The seasonal variation in allergic rhinitis and its correlation with outdoor allergens in Kuwait. Int. Arch. Allergy Immunol..

[63] Al-Dousari A.M., Ibrahim M.I., Al-Dousari N., Ahmed M., Al-Awadhi S. (2018). Pollen in aeolian dust with relation to allergy and asthma in Kuwait. Aerobiologia.

[64] Gautier M., Normand A.-C., Ranque S. (2016). Previously unknown species of *Aspergillus*. Clin. Microbiol. Infect..

[65] Novey H.S., Wells I.D. (1978). Allergic bronchopulmonary aspergillosis caused by *Aspergillus ochraceus*. Am. J. Clin. Pathol..

[66] Guarro J., Kallas E.G., Godoy P., Karenina A., Gené J., Stchigel A., Colombo A.L. (2002). Cerebral aspergillosis caused by *Neosartorya hiratsukae*, Brazil. Emerg. Infect. Dis..

[67] Hakamifard A., Hashemi M., Fakhim H., Aboutalebian S., Hajiahmadi S., Mohammadi R. (2021). Fatal disseminated aspergillosis in an immunocompetent patient with COVID-19 due to *Aspergillus ochraceus*. J. Mycol. Med..

[68] Naidu J., Singh S.M. (1994). *Aspergillus chevalieri* (Mangin) Thom and Church: A new opportunistic pathogen of human cutaneous aspergillosis. Mycoses.

[69] Siqueira J.P.Z., Sutton D.A., Gené J., García D., Wiederhold N., Guarro J. (2018). Species of *Aspergillus* section *Aspergillus* from clinical samples in the United States. Med. Mycol..

[70] Badali H., Shokohi T., Khodavaisy S., Moazeni M., Farhadi M., Nabili M. (2021). Molecular typing of clinical and environmental *Aspergillus fumigatus* isolates from Iran using microsatellites. Curr. Med. Mycol..

[71] Morais S., Toscano C., Simões H., Carpinteiro D., Viegas C., Veríssimo C., Sabino R. (2023). Comparison of multi-locus genotypes detected in *Aspergillus fumigatus* isolated from COVID associated pulmonary aspergillosis (CAPA) and from other clinical and environmental sources. J. Fungi.

[72] Hiel S.J.P., Hendriks A.C.A., Eijkenboom J.J.A., Bosch T., Coolen J.P.M., Melchers W.J.G., Anröchte P., Camps S.M.T., Verweij P.E., Zhang J. (2024). *Aspergillus* outbreak in an intensive care unit: Source analysis with whole genome sequencing and short tandem repeats. J. Fungi.

[73] Chowdhary A., Kathuria S., Randhawa H.S., Gaur S.N., Klaassen C.H., Meis J.F. (2012). Isolation of multiple-triazole-resistant *Aspergillus fumigatus* strains carrying the TR/L98H mutations in the cyp51A gene in India. J. Antimicrob. Chemother..

[74] Chowdhary A., Sharma C., Kathuria S., Hagen F., Meis J.F. (2015). Prevalence and mechanism of triazole resistance in *Aspergillus fumigatus* in a referral chest hospital in Delhi, India and an update of the situation in Asia. Front. Microbiol..

[75] Chen Y., Lu Z., Zhao J., Zou Z., Gong Y., Qu F., Bao Z., Qiu G., Song M., Zhang Q. (2016). Epidemiology and molecular characterization of azole resistance in clinical and environmental *Aspergillus fumigatus* isolates from China. Antimicrob. Agents Chemother..

[76] Dunne K., Hagen F., Pomeroy N., Meis J.F., Rogers T.R. (2017). Intercountry transfer of triazole-resistant *Aspergillus fumigatus* on plant bulbs. Clin. Infect. Dis..

[77] Nakano Y., Tashiro M., Urano R., Kikuchi M., Ito N., Moriya E., Shirahige T., Mishima M., Takazono T., Miyazaki T. (2020). Characteristics of azole-resistant *Aspergillus fumigatus* attached to agricultural products imported to Japan. J. Infect. Chemother..

[78] Denning D.W., Bowyer P. (2013). Voriconazole resistance in *Aspergillus fumigatus*: Should we be concerned?. Clin. Infect. Dis..

[79] Viscoli C., Cornely O.A. (2018). Diagnosis and management of *Aspergillus* diseases: Executive summary of the 2017 ESCMID-ECMM-ERS guideline. Clin. Microbiol. Infect..

