# Supplementary material for: Occurrence of Pathogenic and Allergenic Molds in the Outdoor and Indoor Environment of a Major Hospital and Molecular Epidemiology of Aspergillus fumigatus in Kuwait
Source: J Fungi (Basel). 2025 Jan 21;11(2):83. doi: 10.3390/jof11020083 (PMC11856096; doi:10.3390/jof11020083)
Supplement: Supplementary file 1 [file jof-11-00083-s001.zip › jof-3369220-supplementary.pdf]

Table S1. No. and source of clinical *A. fumigatus* isolates used in this study

Specimen type	No. of *A. fumigatus* isolates
Sputum	58
Endotracheal secretion	25
Bronchoalveolar lavage	8
Ear discharge	5
Pus/wound	5
Peritoneal/pleural/pericardial fluid	5
Nasal/sinus discharge	4
Tissue biopsy	2
Central line tip	1
Nail scrapping	1
Eye swab	1
Urine	1
High vaginal swab	1
**Total**	**117**

Table S2. Nucleotide sequences and specific purpose of primers used in PCR-amplification or DNA sequencing of various genomic regions of *Aspergillus* spp. isolates and the expected sizes of amplicons, where applicable, in base pairs (bp)

Primer name	Nucleotide sequence	Direction	Purpose	Amplicon size* (bp)	Reference
AFUF2	5′-TATGCAGTCTGAGTTGATTATCGT-3′	Forward	*A. fumigatus*-specific PCR amplification of rDNA	318	37
AFUR2	5′-GGCCTACAGAGCAGGTGACAAA-3′	Reverse	*A. fumigatus*-specific PCR amplification of rDNA		37
AFLF2	5′-CTAGTGAAGTCTGAGTTGATTGTAT-3′	Forward	*A. flavus*-specific PCR amplification of rDNA	243	10
AFLR2	5′-CCGGAGAGGGGACGACGA-3′	Reverse	*A. flavus*-specific PCR amplification of rDNA		10
ATEF2	5′-CTTGCAGTCTGAGTGTGATTCTTTGC-3′	Forward	*A. terreus*-specific PCR amplification of rDNA	321	38
ATER2	5′-GGCCTACGGAGCGGAAGACGAA-3′	Reverse	*A. terreus*-specific PCR amplification of rDNA		38
ITS1	5′-TCCGTAGGTGAACCTGCGG-3′	Forward	Panfungal PCR amplification of rDNA	~400-900	39
ITS4	5′-TCTTTTCCTCCGCTTATTGATATGC-3′	Reverse	Panfungal PCR amplification of rDNA		39
ITS1FS	5′- ACCTGCGGAAGGATCATT-3′	Forward	Panfungal DNA sequencing primer for rDNA	N. A.	39
ITS3	5′-TCGCATCGATGAAGAACGCAGC-3′	Forward	Panfungal DNA sequencing primer for rDNA	N. A.	39
ITS4RS	5′- GATATGCTTAAGTTCAGCG-3′	Reverse	Panfungal DNA sequencing primer for rDNA	N. A.	39
ITS2	5′-TCGCTGCGTTCTTCATCGATGC-3′	Reverse	Panfungal DNA sequencing primer for rDNA	N. A.	39
BTUBF	5′-TGGTAACCAAATCGGTGCTGCTT-3′	Forward	PCR amplification of β-tubulin gene fragment	357	40
BTUBR	5′-GCACCCTCAGTGTAGTGACCCT-3'	Reverse	PCR amplification of β-tubulin gene fragment		40
BTUFS1	5′-TAACCAAATCGGTGCTGCTTTCTG-3′	Forward	Sequencing primer for β-tubulin gene fragment	N. A.	40
BTURS	5′-CCTCAGTGTAGTGACCCTTGGC-3'	Reverse	Sequencing primer for β-tubulin gene fragment	N. A.	40
Cmd5	5′-GTCTCCGAGTACAAGGAGGC-3'	Forward	PCR amplification of calmodulin gene fragment	701	40
Cmd6	5′-TCGCCGATRGAGGTCATRACGTG-3'	Reverse	PCR amplification of calmodulin gene fragment		40
CMDFS	5′-TCCGAGTACAAGGAGGCCTTC-3'	Forward	Sequencing primer for calmodulin gene fragment	N. A.	40
CMDRS	5′-GATAGAGGTCATRACGTGRCGCA-3'	Reverse	Sequencing primer for calmodulin gene fragment	N. A.	40
AFCYPPF	5′-AATAATCGCAGCACCACTTC-3'	Forward	PCR primer for tandem repeat in promoter region of *cyp51A*	105 or 139	45
AFCYPPR	5′-TGGTATGCTGGAACTACACCTT-3'	Reverse	PCR primer for tandem repeat in promoter region of *cyp51A*		45
AFCYP51F1	5′-CAGCGGCAGCATTCTGAAACA-3′	Forward	PCR and sequencing primer for *A. fumigatus cyp51A* N-terminal fragment	786	35
AFCYP51R1	5′-CAACAGTATAAAAGTGAAGATAT-3′	Reverse	PCR and sequencing primer for *A. fumigatus cyp51A* N-terminal fragment		This study
AFCYP51F2	5′-AGTTCTTCTTTGCGTGCAGA-3′	Forward	PCR and sequencing primer for *A. fumigatus cyp51A* middle fragment	694	35
AFCYP51R2	5′-GTTCCATATCATGTCTGATTTCT-3′	Reverse	PCR and sequencing primer for *A. fumigatus cyp51A* middle fragment		35

AFCYP51F3	5′-ATGAGGTCAATCTACGTTGA-3′	Reverse	PCR and sequencing primer for *A. fumigatus cyp51A* C-terminal fragment	911	35
AFCYP51R3	5′-CGAGGGGCTGAATTAAGTATAA-3′	Reverse	PCR and sequencing primer for *A. fumigatus cyp51A* C-terminal fragment		This study

*Amplicon sizes (in base pairs, bp) are based on various combinations of forward primers with their reverse primer. The amplicon sizes for rDNA with panfungal primers ITS1 and ITS4 vary among different fungal species due to variations in the length of internal transcribed spacer-1 and internal transcribed spacer-2 regions. N. A., not applicable
